# Investigation of neuronal pathfinding and construction of artificial neuronal networks on 3D-arranged porous fibrillar scaffolds with controlled geometry

**DOI:** 10.1038/s41598-017-08231-3

**Published:** 2017-08-10

**Authors:** Dongyoon Kim, Seong-Min Kim, Seyeong Lee, Myung-Han Yoon

**Affiliations:** 1 School of Materials Science and Engineering, Gwangju Institute of Science and Technology, 123 Cheomdan-gwagiro, Buk-gu, Gwangju, 61005 Republic of Korea; 2 Korea Institute of Energy Research (KIER), 152 Gajeong-ro, Yuseong-gu, Daejeon, 34129 Republic of Korea

## Abstract

Herein, we investigated the neurite pathfinding on electrospun microfibers with various fiber densities, diameters, and microbead islands, and demonstrated the development of 3D connected artificial neuronal network within a nanofiber-microbead-based porous scaffold. The primary culture of rat hippocampal embryonic neurons was deposited on geometry-controlled polystyrene (PS) fiber scaffolds while growth cone morphology, neurite outgrowth patterns, and focal adhesion protein expression were cautiously examined by microscopic imaging of immunostained and live neuronal cells derived from actin-GFP transgenic mice. It was demonstrated that the neurite outgrowth was guided by the overall microfiber orientation, but the increase in fiber density induced the neurite path alteration, thus, the reduction in neurite linearity. Indeed, we experimentally confirmed that growth cone could migrate to a neighboring, but, spatially disconnected microfiber by spontaneous filopodium extrusion, which is possibly responsible for the observed neurite steering. Furthermore, thinner microfiber scaffolds showed more pronounced expression of focal adhesion proteins than thicker ones, suggesting that the neuron-microfiber interaction can be delicately modulated by the underlying microfiber geometry. Finally, 3D connected functional neuronal networks were successfully constructed using PS nanofiber-microbead scaffolds where enhanced porosity and vertical fiber orientation permitted cell body inclusion within the scaffold and substantial neurite outgrowth in a vertical direction, respectively.

## Introduction

Electrospinning is a simple but versatile technique for fabricating micro/nanofibers, and both single fibers and entangled fibrillar scaffolds have been extensively exploited for energy storage devices^1, 2^, sensors^3, 4^, and biomedical applications^5–9^. Beside the relative simplicity and scalability in fabrication, one common reason for its popularity is that electrospun fibers exhibit large porosity as well as high surface area-to-volume ratio, which are not straightforward to generate by other conventional bottom-up or top-down fabrication methods. Particularly, structural parameters of electrospun fiber scaffolds (i.e., fiber diameter and pore size) are typically in the range of 0.1 to 10 micrometers, which is comparable to the length scale of single cell processes such as cellular alignment, migration, proliferation, etc^10–14^. In fact, many researchers have used electrospun fibers as a culture platform for studying various types of cells including stem cells. Previous studies showed that the mechanical^15, 16^, geometrical^17^, and chemical properties^18, 19^ of electrospun fiber scaffolds can affect several different types of apparent cell behaviors and protein expression levels. After it was reported that neuronal processes (i.e., neurites) can follow the directionality of underlying micro/nanofibers, electrospun fibers have been widely used as an artificial culture substrate for various biological and biomedical studies^20–22^. In particular, peripheral nerve guidance and combinatorial approaches based on biodegradable polymer fibers have shown their strong potential for treating human nervous systems in the future^8, 23^.

Apart from neural engineering and biomedical applications, electrospun fibers can be employed as a substrate platform beneficial for studying the fundamental aspects of neurite outgrowth not only in the peripheral but also central nervous system (CNS), since cellular contact points are confined on the surface of a pseudo-one-dimensional (1D) structure of electrospun micro/nanofiber. Therefore, it is conceivable that, in principle, neurite directionality and synaptic connection could be designed *de novo* by the overall geometry of electrospun fiber scaffolds used for neuronal culture. Several research groups have already demonstrated that the axonal guidance is typically in parallel with the electrospun fiber alignment^24–26^. On the contrary, it was also reported that axonal guidance could occur in the direction perpendicular to aligned fibers^27^. These apparently contradictory results imply that there exists a certain possibility that neurite pathfinding and outgrowth may be guided not only by ‘static’ environmental factors, i.e., the underlying micro/nanofiber directionality, but also by ‘dynamic’ cellular factors, i.e., growth cone movement and its interaction with extracellular structures. Nonetheless, most of the previous studies on neuronal culture on electrospun fibrillar scaffolds focused on the guidance of peripheral nerve growth on the parallelly-aligned electrospun fibers, which can be prepared by electrospinning on rotating metallic drum^28^ or parallelized collecting electrodes^29^. Note that these reports mainly described the formation of neuronal circuit along the aligned direction of underlying fiber, where the fiber alignment was considered as a critical factor for efficient generation of neuronal circuit. Unfortunately, there exist only few studies on CNS-derived neurons on electrospun micro/nanofibers although such a fibrillary network can be employed to define 3D artificial neuronal networks which could be useful in the field of fundamental neuroscience and/or developmental neurobiology. Moreover, it has not been clarified how neurite outgrowth of CNS neurons can be modulated by structural parameters of underlying fibrillar scaffolds, for instance, fiber diameter, density, porosity, etc.

In this study, we fabricated various types of free-standing fibrillar scaffolds which consist of entangled electrospun polystyrene (PS) micro/nanofibers with controlled geometric parameters, and investigated growth cone dynamics and neuronal cell morphology by fluorescence microscopy in combination with time-lapse live imaging. Particularly, electrospun fibers were visualized by confocal reflection microscopy (CRM), which permits the overlay of neuronal processes and micro/nanofibers, thus, the microscopic examination of neuron-fiber interaction in terms of growth cone morphology and focal adhesion expression using fibrillar scaffolds with distinct fiber density and diameter. Finally, 3D connected artificial neuronal networks were constructed within a nanofiber-microbead-based porous scaffold where the enhanced porosity and vertical fiber orientation enabled cell body inclusion within the scaffold and substantial neurite outgrowth in a vertical direction, respectively.

## Results

### Preparation of fibrillary scaffolds with controlled geometry and microbead inclusion via electrospinning

**Figure 1:**
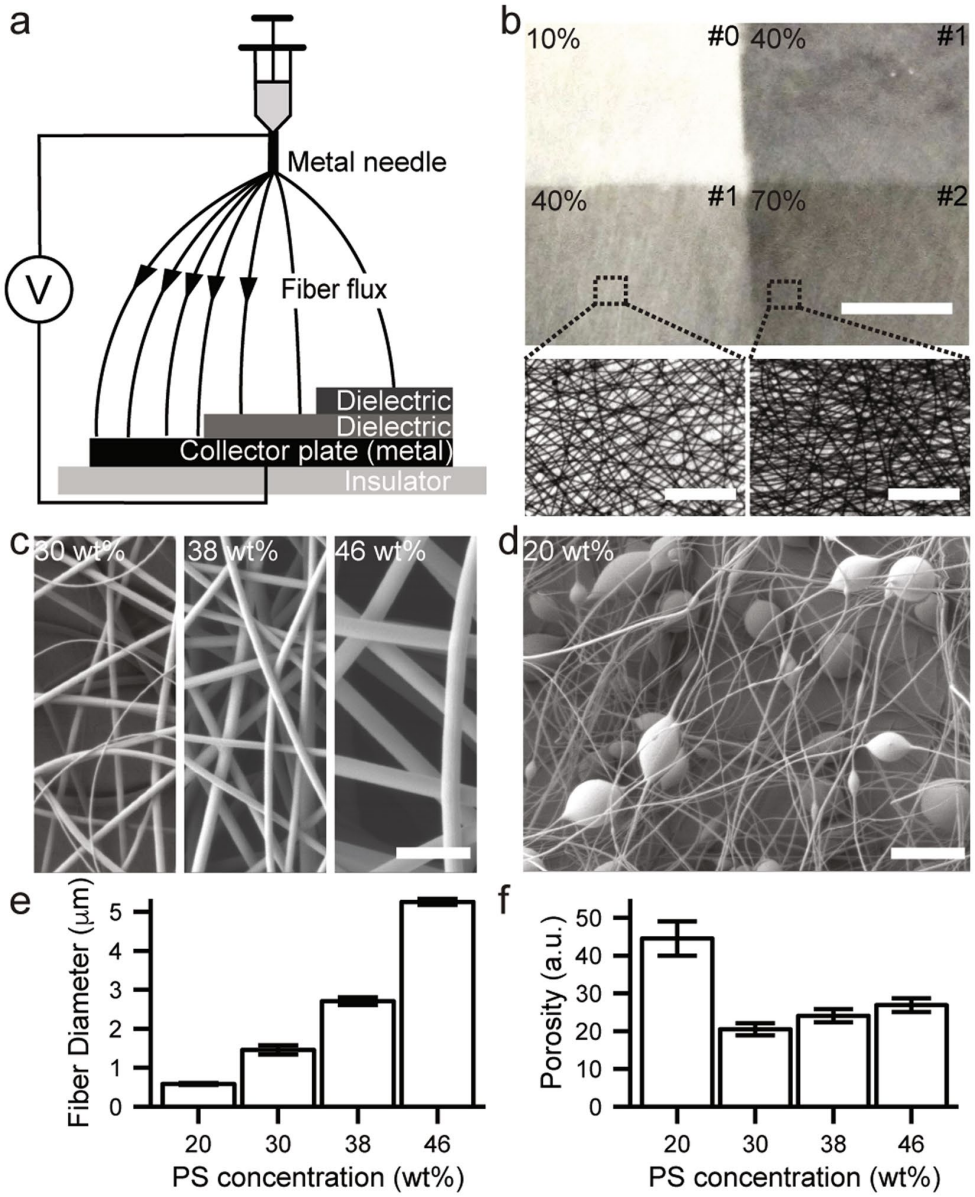
(**a**) A schematic illustration of electrospinning with controlled structural parameters such as fiber density. (**b**) The optical micrograph of density-varied electrospun fibers under low (upper, scale bar: 500 μm) and high magnifications (lower, scale bar: 100 μm). The upper-right metrics represent the number of stacked paraffin films. (**c**) and (**d**) Scanning electron microscopy (SEM) images of diameter-controlled electrospun fibers and microbead-incorporated nanofiber mesh (scale bars: 20 μm). (**e**) and (**f**) Plots of fiber diameter (n = 25, 30, 30 and 30 fibers for 20, 30, 38 and 46 wt% of PS concentration, respectively) and porosity depending on the PS concentration of precursor solution, respectively. All error bars denote standard error of the mean.

Figure 1(a) shows a schematic diagram of electrospinning apparatus employed in this study. Fiber density, diameter, and microbead inclusion were judiciously controlled by adjusting the experimental condition of electrospinning. First, the areal density of electrospun micro/nanofibers was altered by inserting paraffin films between a metal needle and a collector plate. The increase in overall dielectric film thickness reduced the local flux of electrospun fibers, leading to the decrease of resultant fiber density, while the fiber diameter was nominally fixed as shown in the corresponding optical microscopy images (Fig. 1(b)). Next, the diameter of electrospun fiber was manipulated by varying the concentration of PS solution. Figure 1(c) and (e) show that less concentrated PS solutions yield fibers with thinner diameters. This result can be attributed to the reduced viscoelasticity of PS solution, leading to the facilitated deformation of a polymer solution drop at the given high electric field, thus, the decreased diameter of electrospun fiber. Interestingly, this proportionality ceased around 20 wt% of PS concentration, where fiber diameters were not relatively uniform as shown in Fig. 1(d). The resultant electrospun scaffold contained a mixture of microscale beads and nanofiber threads^30^, which showed much higher porosity (i.e., larger interfiber spacing) than electrospun microfibers prepared using the solutions of higher ( > 20%) PS concentration (Fig. 1(f)).

### Examination of growth cone morphology and neurite directionality

**Figure 2:**
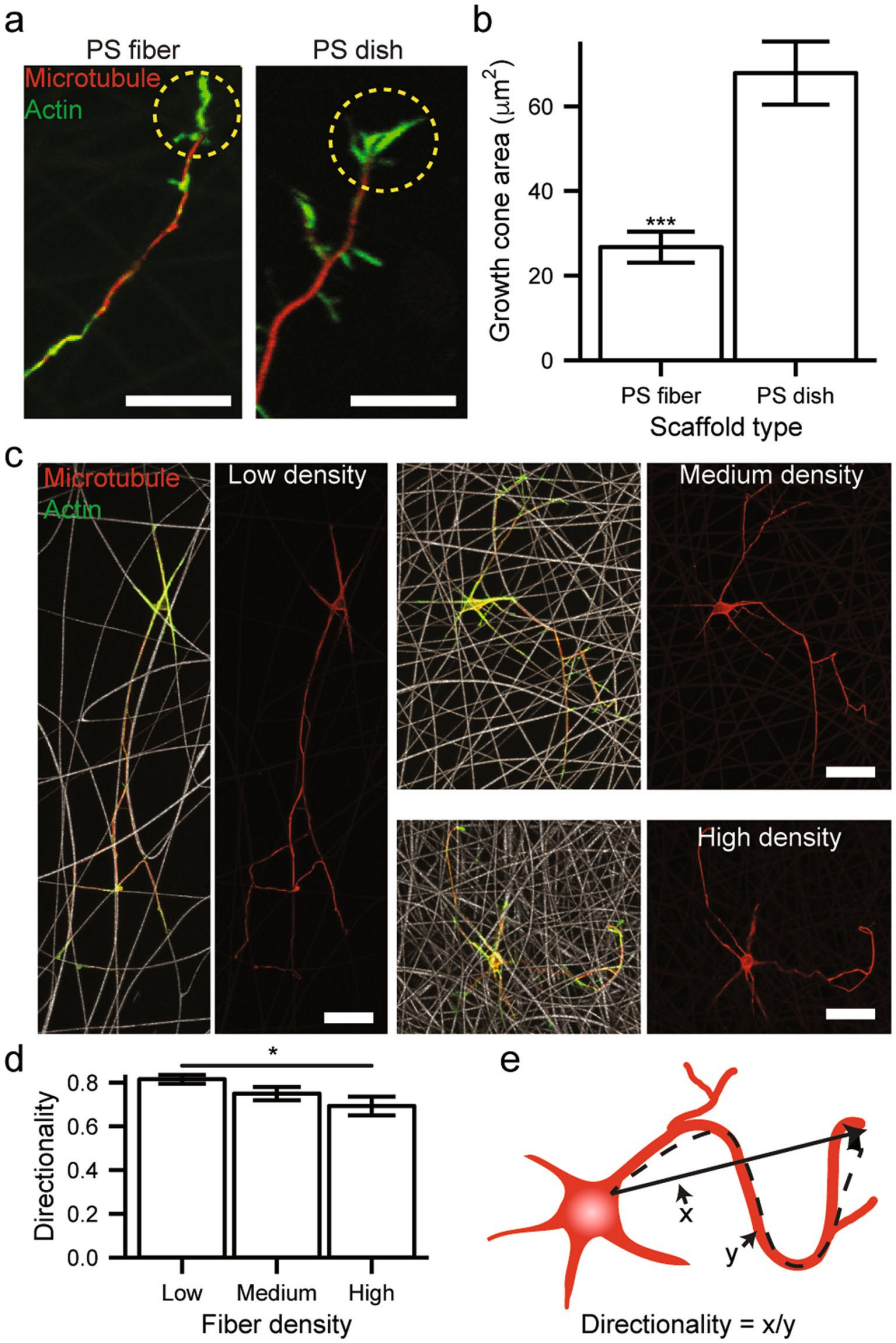
(**a**) Laser scanning confocal microscopy images of immunostained growth cones of 5-DIV neurons on electrospun PS fibers and PS culture dish (scale bar: 20 μm). Microtubules and actin filaments were labeled with tuj1 (red) and phalloidin (green), respectively. (**b**) Statistical distributions of growth cone area on each substrate (***P < 0.001). (**c**) Fluorescence microscopy images of neurons cultured on electrospun fibers with three different densities (i.e., low, medium, high). Microtubules and actin filaments were labeled with tuj1 (red) and phalloidin (green), respectively (scale bar: 20 μm). (**d**) Statistical distributions of neuronal directionality (i.e., straightness) depending on electrospun fiber coverage (*P < 0.05). (**e**) A schematic of neuronal directionality definition (the ratio of vectorial length over trajectory length). All error bars denote standard error of the mean.

First of all, the primary culture of rat hippocampal embryonic (E18) neurons was conducted on various fibrillar scaffolds after poly-D-lysine treatment and the biocompatibility was examined (Fig. S1, Supporting Information). Note that the viabilities of neuronal cells on all PS fibrillary scaffolds were comparable to that on flat PS dish at 3 DIV although the prolonged culture was more viable on flat dish, possibly, due to the facile removal of undesired cell debris during the medium exchange. Next, for the detailed investigation of neurite outgrowth dynamics on a geometry-controlled pseudo-1D microfiber, growth cone or a leading edge of axon, which determines neuronal elongation in response to external stimuli, was visualized on PS dish and microfibers via actin labeling. Figure 2(a) and (b) reveal that there exists a substantial difference in growth cone morphology between two substrates. In the case of electrospun microfiber mesh, most of the growth cones were concentrated at the distal part of neurites with relatively small area compared with those on a flat PS dish. We argue that the cylinder-like electrospun fiber structure confines most of the pathfinding activities of growth cone along the underlying fiber direction, leading to the reduction in actin coverage area. Next, after primary embryonic neurons were cultured on porous fibrillar scaffolds with three different fiber densities (i.e., high, medium, and low) for 7 DIV, cells were immunostained with a microtubule marker, while electrospun microfibers themselves were simultaneously visualized with CRM. As shown in Fig. 2(c) and (d), the relatively high fiber density mesh exhibited the statistically meaningful mitigation in neurite outgrowth (linear) directionality (see Fig. 2(e) for its schematic definition). This result suggests that the more frequent crossing-over among electrospun fibers, especially, at the high-fiber-density scaffold, can induce neurite to switch to another neighboring fiber, leading to the decrease in the overall neurite directionality.

### Neuronal locomotion on fibrillary scaffold

**Figure 3:**
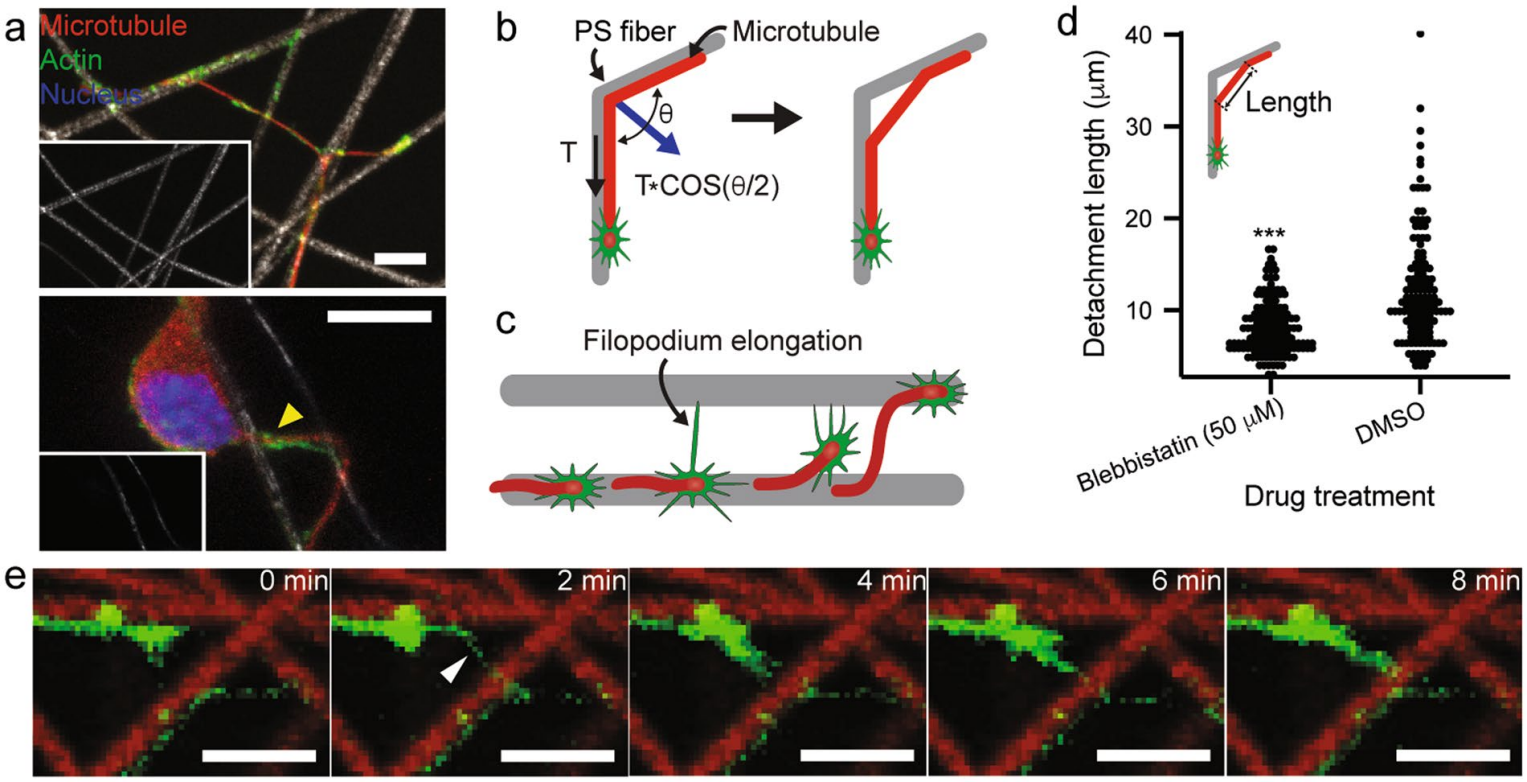
(**a**) Immunofluorescence images of 5 DIV neurons on electrospun PS fiber mesh showing neurites hanging over fibers. Microtubules and actin filaments were labeled with tuj1 (red) and phalloidin (green), respectively (scale bar: 10 μm). (**b**) and (**c**) Two proposed scenarios on neurite detachment from the fibrillary substrate via growth cone’s traction force and filopodium elongation. (**d**) Comparison of the length of detached neurite from the underlying fiber between 50 μM blebbistatin- and DMSO-treated (control) neurons. The average diameter of used PS fibrillary scaffold is 2.3 μm and the detached neurite length was extracted from the fluorescence microscopy images of neurons (5 DIV) immunostained with tuj1 (***P < 0.001, the total cell number is 150 for each condition). (**e**) A series of time-lapse confocal microscopy images of actin-GFP expressing mouse hippocampal neuron (green) and confocal reflection microscopy-revealed electrospun fibers (red) (scale bar: 10 μm).

To further understand the locomotion and steering of growth cone on spatially-separated micro/nanofibers, paraformaldehyde-fixed neurons were carefully monitored, particularly, on curved neurite regions. The microscopic images of immunostained neurons (Fig. 3(a)) show two distinct types of freestanding curved neurites hanging over spatially-separated microfibers: crossing fibers with an abrupt angle vs. parallel fibers with a small spacing. First, neurites could be partially detached from crossing microfibers with an abrupt angle (*θ*) (Fig. 3(a) upper). As depicted in Fig. 3(b), the neurite detachment could be assisted by the traction force (*T*_*trac*_) of growth cone because the detaching force is proportional to *T*_*trac*_ *cos(θ/2)* while the elastic property of microtubule also impedes its bending^31^. Second, neurite could spontaneously migrate to another microfiber which is in parallel with the direction of neurite outgrowth with relatively small spacing (Fig. 3(a) lower). In this case, the growth cone’s switch to another fiber could be enabled by filopodium elongation as schematically depicted in Fig. 3(c). To verify the roll of traction force during this course, the non-muscle myosin II inhibition assay with blebbistain was conducted. As shown in Fig. 3(d) inset, the length of detached (free-standing) neurite is compared between 50 μM blebbistatin- and DMSO-treated (control) neurons. The total numbers of free-standing neurites of blebbistatin- and DMOS-treated neurons are 158 and 145, respectively. Remarkably, the majority of neurons where the expression of non-muscle myosin II was inhibited by blebbistatin treatment, exhibited relatively short free-standing neurites compared to the control group. This evidence suggests that the detachment of neurites at the given situation could be assisted by traction force. To investigate the existence of spontaneous growth cone migration, time-lapse fluorescence microscopy was performed on actin-GFP transgenic mice derived embryonic hippocampal neurons. As demonstrated in Fig. 3(e) (see also Fig. S2, Supporting Information), growth cone was actively searching for its pathway by scanning not only the surface of electrospun fibers but also the substrate-free interfiber region. Occasionally, the spontaneous elongation of filopodium leads to the eventual migration to another fiber through the interfiber void (i.e., no fiber region). The above-mentioned behaviors of neuronal growth cone on the electrospun fibrillar scaffold are very similar to those of worm on tree branches, implying that growth cone dynamic is mechanically robust and, therefore, can maintain its structural integrity even in the absence of adhesion support.

### Investigation of focal adhesion protein expression

**Figure 4:**
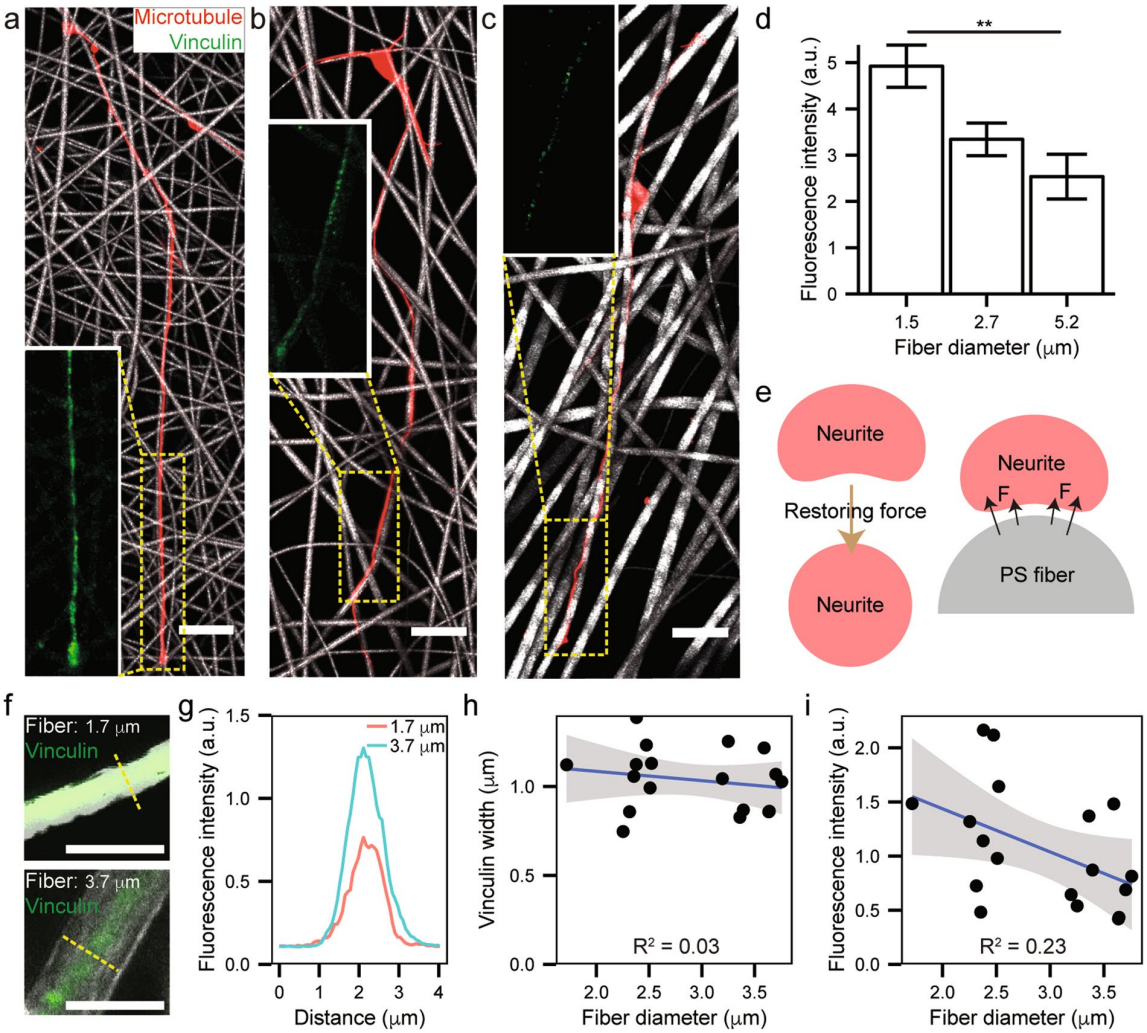
(**a**), (**b**) and (**c**) Fluorescence microscopy images of vinculin-expressing neurons (7 DIV) depending on PS fiber diameter (1.5, 2.7, and 5.2 μm, respectively). Red and green represent microtubule and vinculin, respectively (scale bar: 20 μm). (**d**) A statistical distribution of vinculin fluorescence intensity shown in Fig. 4a–c (**P < 0.01). (**e**) Proposed mechanism on fiber diameter-dependent vinculin expression. all error bars denote standard error of the mean. (**f**) Laser scanning confocal fluorescence microscopic images of neurites stained with vinculin (green) on PS fiber scaffolds (white; upper 1.7 μm and lower 3.7 μm of diameters) at the 7-DIV neuronal culture. Scale bars denote 5 μm. (**g**) Cross-sectional fluorescence intensity profiles of vinculin as indicated by yellow lines in (**f**). (**h**) Distributions of the lateral width of vinculin expression depending on the underlying fiber diameter. (**i**) Distributions of fluorescence intensity of vinculin depending on the underlying fiber diameter.

In parallel, the adhesion of neuronal cells onto the surface of electrospun micro/nanofibers was also investigated. Focal adhesion is a protein complex which forms a junction between cytoskeleton and extracellular matrix. The spatial coverage and expression level of focal adhesion-related protein complexes are influenced by mechanical rigidity, ligand density, and type of extracellular matrix^32–37^. For instance, the expression level of vinculin, a type of focal adhesion proteins, is determined by the interaction between myosin motor-mediated traction force and elastic modulus of adhesion site to integrin^38^, and a stiff substrate triggers strong myosin traction force so that the vinculin expression level is substantially increased^33^. Remarkably, Fig. 4(a)–(d) show that the increase of electrospun fiber diameter leads to the reduction of focal adhesion protein level. In contrast, the neurons cultured on thinner electrospun fiber mesh exhibited higher fluorescence intensity of vinculin labeling throughout the whole axon than those cultured on thicker electrospun fiber mesh. One may expect the reduced vinculin level on thinner microfibers due to their low stiffness compared to thicker microfibers^39^ (Fig. S3, Supporting Information). Note that the modulation of fiber stiffness is more effectively controlled by varying its diameter rather than intrinsic modulus^39–41^. To address this apparent contradiction, we speculate that the self-strained focal adhesion might be involved especially on thinner fibers as schematically shown in Fig. 4(e). Due to the elastic modulus of neurite itself, its adhesion to the curved surface of microfiber generates the restoring force of neurite cylinder itself at the interacting surface between neurite and electrospun fiber. Therefore, if electrospun microfiber was much thicker than the curvature of neurite, the tension could be more equally distributed throughout the focal adhesion complex, and the force exerted on integrin could be moderated, leading to the decrease in vinculin expression. In fact, the detailed vinculin expression was carefully examined to understand the correlation between neurite contact area and fiber diameter (Fig. 4(f) to (i)). The statistical analyses of spatial distributions and intensity profiles of vinculin at different fiber diameters indicate that while the underlying fiber diameter minimally affects the contact area or lateral spreading of neurite, the vinculin expression is enhanced by the reduction of fiber thickness.

### Construction of 3D artificial neuronal network on highly porous nanofiber-microbead scaffold

**Figure 5:**
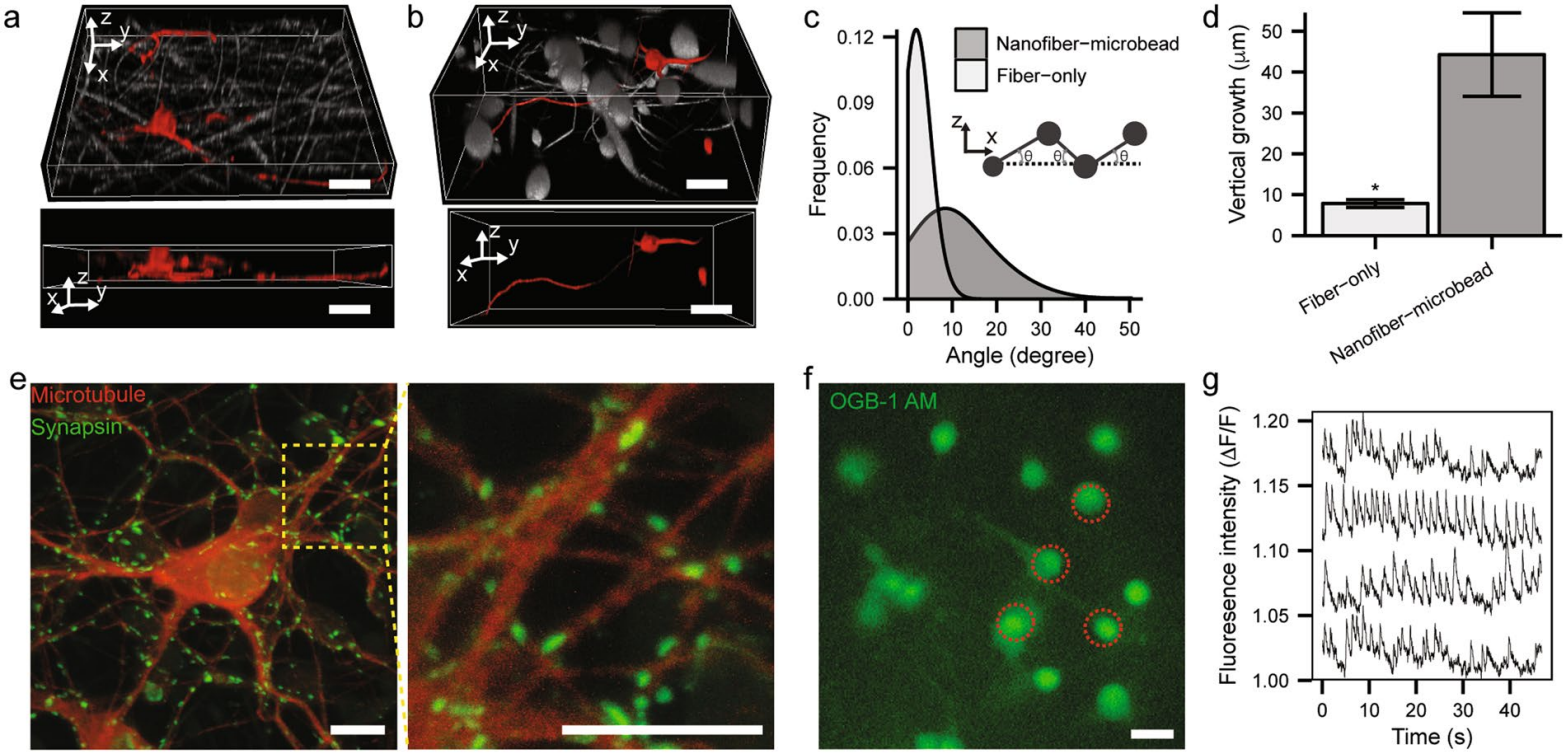
3D reconstructed confocal microscopy images of neuronal culture (5 DIV) in (**a**) electrospun fiber-only and (**b**) nanofiber-micro bead mesh (scale bar: 10 μm). (**c**) Normalized statistical distributions of off-horizontal angle of fiber orientation in fiber-only (white) and nanofiber-microbead scaffolds (gray) (see also the inset scheme). (**d**) Averaged overall vertical growth of neurites on fiber-only (white) and nanofiber-microbead scaffolds (gray) (*p < 0.05). (**e**) Fluorescent images of immunostained neurons (14 DIV) on nanofiber-microbead mesh scaffold. Red and green colors represent microtubule and synapsin, respectively (scale bar: 10 μm). (**f**) A fluorescence image of neurons (14 DIV) cultured on the nanofiber-microbead scaffold after staining Oregon Green 488 BAPTA-1 AM (scale bar: 10 μm). (**g**) Representative traces of quantified calcium signaling (ΔF/F) from cells indicated by red circles in (**f**).

Until now, we have investigated neuronal dynamics demonstrated on electrospun fiber surface without considering vertical neuronal outgrowth. Typically, the overall fiber orientation in the conventional electrospun micro/nanofiber scaffold is in parallel with the collecting metal plate. Therefore, neurons cultured on electrospun fibrillary scaffolds mostly display a majority of cell bodies sitting on top of mesh and neurite extension confined in parallel with the horizontal plane, however, rarely exhibit neurite outgrowth in a vertical direction (Fig. 5(a)). In contrast, the neuronal culture on electrospun PS nanofiber-microbead scaffold exhibited several features of 3D-like neuronal culture, for instance, cell inclusion inside the scaffold and vertical neurite outgrowth (Fig. 5(b)), although the neuronal directionality along the fiber was not significantly different between fiber-only and nanofiber-microbead scaffolds (Fig. S4, Supporting Information). Note that in comparison with the electrospun fiber-only scaffold, the incorporation of microbeads into fibrillar mesh enables both enlarged free volume and enhanced vertical fiber orientation (Fig. 5(c)), resulting in cell body penetration inside scaffold and vertical neurite guidance (Fig. 5(d)) with large inter-fiber spacing, respectively. Finally, Fig. 5(e)–(g) shows the expression of synaptic proteins (e.g., synapsin) and the activity of neuronal firing (e.g., calcium signaling) on bead-nanofiber scaffold. Furthermore, the neurons on microbead-nanofiber scaffold showed the distinct functional activity compared to those on PS dish (Fig. S5, Supporting Information)^42–45^. These results suggest the future potential of the 3D-like neuronal culture for artificial neuronal network formation and other related studies.

## Discussion

Due to the contact cue from electrospun fibers, the pathfinding process of the cultured neuron is significantly influenced by the supporting fiber geometry. However, as shown in our research, such a contact-derived guidance effect can be moderated by increasing the fiber density up to a certain degree because the spontaneous elongation of filopodium can induce the migration of grown cone, thus, neurite to another neighboring fiber. To our knowledge, we report the experimental verification of such through-space neurite migration for the first time. Therefore, neurite orientation is not only affected by scaffold geometry ^46, 47^, but also by its own active dynamics. This finding may be exploited for varying neurite outgrowth directions beyond the contacting fiber orientation and, thereby, enriching the morphology/connectivity of resultant artificial neuronal network, which is one of the most important factors to determine the neuronal network function. In the peripheral nervous system, directionality is one of the important factors for the effective propagation of nerve signal. In the case of CNS, however, network connectivity has been attributed to the origin of higher-order brain functions^48^. Therefore, a variety of *in vitro* 3D models emulating *in vivo* neuronal networks have been proposed by many researchers. As shown in Fig. 5(a), electrospun micro/nanofiber scaffolds typically confine the overall neuronal network within ~10 μm thick mesh from the top surface of culture because most of the fibers exhibit the horizontal orientation as well as low porosity (Fig. 1(c) and (f)). In contrast to the conventional fiber-only scaffolds with 2D-like network morphology, the electrospun nanofiber-microbead scaffold not only contains more off-horizontal fiber orientations (Fig. 5(c)) but also exhibit substantially increased porosity (Fig. 1(f)) compared to fiber-only scaffolds. Indeed, microbead incorporation assisted the inclusion of neuronal bodies below the culture surface (Fig. 5(b)) and the enhanced vertical (z-axis) growth of neurite down to 44 μm from the top culture surface (Fig. 5(d)). Furthermore, it is noteworthy that the enlarged porosity permitted the co-culture of neurons and glia with larger number of cells inside the scaffold (see Fig. S6 and Movie S1–3, Supporting Information). Considering that in the real brain tissue, there exist more supporter cells (i.e., glia) than neurons^48^, such a highly-packed neuronal co-culture could be beneficial for emulating the brain tissue environment, thus, addressing the fundamental neuroscience questions.

## Conclusions

In this work, the investigation of neuronal pathfinding on geometry-defined fibrillar scaffolds revealed that topographical factors of electrospun fibers significantly influence neuronal cell morphology and outgrowth pattern. We observed that neurites outgrowth followed the overall microfiber direction, but, the increase in fiber density reduced the neurite linearity, in which course growth cone promoted neurite migration to a spatially-separated fiber. Moreover, we discovered that neurons cultured on lower-diameter fibers exhibited higher expression of vinculin, implying that the geometrical parameters of fibers can be used for controlling the adhesion of neurites on underlying scaffolds. Finally, the artificial 3D neuronal network was successfully constructed using electrospun nanofiber-microbead scaffolds, which permitted substantially enhanced porosity, thus, cell body inclusion inside the scaffold and, simultaneously, noticeable neurite penetration in a vertical direction. Our results suggest that electrospun fibrillar scaffolds with well-defined fiber geometry and microbead inclusion may function as a versatile culture platform for various types of artificial 3D neuronal networks.

## Materials and Methods

### Fabrication and characterisation of polystyrene electrospun fibers

Polystyrene (PS) at 20 wt%, 30 wt%, 38 wt%, and 46 wt% (Sigma-Aldrich, M_w_ = 280,000) in DMF (Sigma-Aldrich, 99.8%) was stirred overnight. Dissolved transparent PS precursor was loaded on 3 mL syringe and 21 G metal needle (BD) was retracted in front of the syringe. The syringe was fixed in a single syringe infusion pump (KD Scientific) and placed on top of collecting metal sheet. DC high voltage generator (Chungpa EMT) supplied voltage through sheathed conducting cable that was connected to the metal needle and collecting grid. The supplying voltage and infusion speeds were 18 kV and 0.1 mL, respectively. The distance between the metal needle and collecting metal grid was 20 cm. After the spinning process, the PS electrospun fiber was treated with oxygen plasma treatment for 1 min to make the surface of electrospun PS fiber hydrophilic. The electrospun PS fibers were immersed into poly-D-lysine (Sigma-Aldrich) solution (0.1 mg/mL), dissolved in 50 mM of boric acid hydroxide (Sigma-Aldrich) solution overnight, and then washed with distilled water intensively before cell seeding. Ethanol was used as a swelling liquid to measure the swelling ratio due to the amphiphilic property and low surface energy, which minimizes overestimation of swollen liquid. The diameter and surface morphology of PS fiber were characterized by a field-emission scanning electron microscope (FE-SEM, JEOL 6301 F, Japan). Platinum (Pt) coating was needed prior to SEM characterization to avoid image distortion.

### Primary culture of rat hippocampal embryonic neurons

Rat embryos (embryonic day 18, E18) was sacrificed and the hippocampal region was dissected in HBSS (Hank’s buffer salt solution, Sigma-Aldrich) medium under a stereomicroscope. The hippocampal tissue was then dipped into 140 μL of papain (Worthington Biochem, Corp) and 30 μL of DNase (30 units, Invitrogen) in 20 mL of HBSS. After 20 min of incubation, HBSS was removed and 10% (v/v) FBS containing HBSS was added to block enzymatic activity. The medium was replaced with HBSS and dissociated mechanically by pipetting in 30 μL DNase (30 units) in 2 mL HBSS. Pipetting was executed about 50 times until there was no visible supernatant. The dissociated hippocampal neuron was seeded on poly-D-lysine coated electrospun PS fiber with plating medium (Invitrogen) with 10% FBS. After 1 hour of incubation, the medium was changed to neurobasal medium (Invitrogen). The culture medium was replaced every three days. All the animal experiments were and protocols were approved by the Institutional Animal Care Use Committee of the School of Life Sciences, Gwangju Institute of Science and Technology and carried out in accordance with their approved guidelines (IACUC GIST- 2014-06).

### Immunocytochemistry and imaging

Neurons cultured *in vitro* for 5 days (5 DIV), 7 days (7 DIV), and 14 days (14 DIV) on electrospun PS fiber were fixed using paraformaldehyde for 30 min and treated with Triton X-100 (Sigma-Aldrich) for 10 min. Bovine serum albumin (BSA, 10%) was used to block the non-specific binding for 40 min. Cellular proteins for β3-tubulin, axon, dendrite, vinculin, synaptophysin, and GFAP were stained with rabbit anti-tuj1 (1:40001, Abcam), mouse monoclonal anti-tau (1:200, SCBT), rabbit polyclonal anti-MAP2 (1:200, SCBT), mouse monoclonal anti-vinculin (1:200, SCBT), rabbit polyclonal anti-synaptophysin (1:1000, Abcam ab68851), and chicken polyclonal anti-GFAP (1:1000, Abcam ab4674) in 3% BSA, respectively. The samples were stored at 4 °C for 1 day and then washed with PBS three times for 15 min each. The following secondary antibodies were used- anti-rabbit Alexa Fluor 405 (1:10001, Invitrogen), anti-mouse Alexa Fluor 488 (1:1000, Invitrogen), anti-rabbit Alexa Fluor 488 (1:1000, Invitrogen), anti-mouse Alexa Fluor 543 (1:1000, Invitrogen), anti-rabbit Alexa Fluor 543 (1:1000, Invitrogen), anti-mouse Alexa Fluor 635 (1:1000, Invitrogen), and anti-chicken Alexa Fluor 635 (1:1000, Invitrogen). The actin filaments were stained with Alexa Fluor 488 - conjugated phalloidin (1:200, Lonza Group Ltd). The sample was stored at 4 °C for 12 hours and then washed with PBS three times for 15 min each. The nuclei were stained immediately before imaging with 0.1 μg/mL of DAPI (Invitrogen).

### Imaging and data analysis

Upright confocal microscope (Olympus BX61WI) was used to investigate the neuronal morphology and protein expression. Confocal reflection mode (CRM) revealed non-labelled electrospun PS fiber by opening excitation filter. All confocal images were taken using 20× water dipping objective lens (XLUMPLFLN-W, Olympus). The synaptophysin was imaged by 60× water dipping lens (LUMFLN 60XW, Olympus). The fluorescence intensity was calculated by subtracting mean fluorescence of background reading from integrated fluorescence intensity of the selected cell and then multiplying it with the area of selected cell. Measurement of actin distribution, fluorescence intensity, neuronal straightness, fiber diameter, and fiber coverage was performed using ImageJ (NIH). The fiber coverage for low density, middle density, and high density correspond to 10~35%, 35~55% and 55~95%, respectively. The fiber density was calculated by measuring covering ratio of electrospun fiber from the CRM image. Statistical significance was evaluated by pairwise-t-test method built in statistical programming language R. All of the error bars denotes standard error of the mean.

### Live cell imaging of rat hippocampal embryonic neurons on electrospun PS fiber

To interrogate neuronal dynamics on PS fiber, hippocampal neurons from actin-GFP transgenic mice were employed. The primary culture protocol used was the same as that for rat hippocampal neuron culture. The live cell imaging for neuronal dynamics was performed under live cell imaging chamber (LCI, South Korea) installed on the upright confocal microscope.

### Calcium imaging

5 μg of Oregon Green™ 488 BAPTA-1 was dissolved in 20% (v/v) Pluronic F-127 containing 1 μL of dimethyl sulfoxide (DMSO) solution and, then, mixed with neurobasal medium. Subsequently, the cell-seeded scaffold was transferred into the resultant solution. fter 10-min incubation, the calcium dye-containing medium was replaced by the original culture medium and incubated for 10 minutes before imaging. The time-lapse calcium imaging was executed inside the live cell imaging chamber (LCI, South Korea). The frame rate of image recording was either 22 or 33 fps (frames-per-second) depending on the signal quality at minimum light intensity. The fluorescent calcium signals (ΔF/F) were calculated using the following equation.

$$\frac{{\rm{\Delta }}F}{F}=\frac{current\,fluorescence\,intensity-lowest\,fluorescence\,intensity}{lowest\,fluorescence\,intentsity}.$$

### Live/dead assay

2 μL of ethidium homodimer-1 solution (Invitrogen) and 1 μL of calcein AM solution (Invitrogen) were mixed with 3 mL of PBS, and the final mixture was transferred to the culture dish containing the designated neuronal culture, immediately after the existing medium was removed.

### Blebbistatin assay

One day after cell seeding, the culture medium was replaced by the blebbistatin (Sigma-Aldrich)-containing medium (50 μM), and the control samples were treated with 1 μL amount of DMSO. The drug treatment was resumed until cell fixation.

## Electronic supplementary material

Movie S1

Movie S2

Movie S3

Supporting Information
