# Supplementary material for: Investigation of neuronal pathfinding and construction of artificial neuronal networks on 3D-arranged porous fibrillar scaffolds with controlled geometry
Source: Sci Rep. 2017 Aug 10;7:7716. doi: 10.1038/s41598-017-08231-3 (PMC5552865; doi:10.1038/s41598-017-08231-3)
Supplement: Supplementary file 4 — Supporting Information [file 41598_2017_8231_MOESM4_ESM.doc]

Supporting Information

**Investigation of neuronal pathfinding and construction of artificial neuronal networks on 3D-arranged porous fibrillar scaffolds with controlled geometry**

Dongyoon Kim, Seong-Min Kim, Seyeong Lee, and Myung-Han Yoon*

School of Materials Science and Engineering, Gwangju Institute of Science and Technology, 123 Cheomdan-gwagiro, Buk-gu, Gwangju 61005, Republic of Korea

E-mail: mhyoon@gist.ac.kr


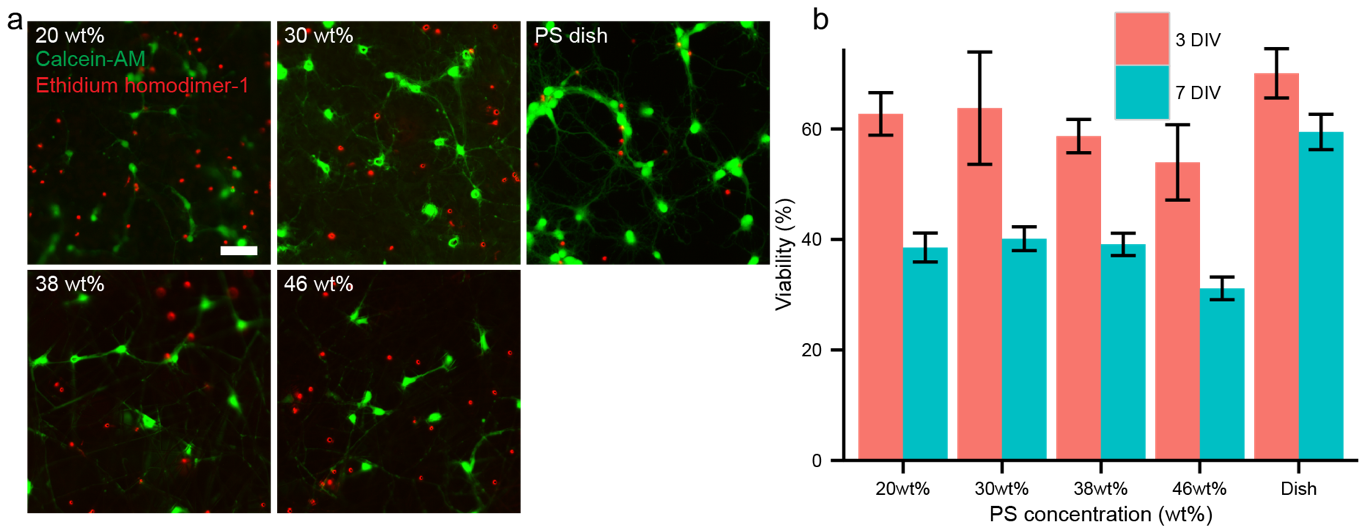


**Figure S1**. (a) Fluorescent images of primary neuronal cells cultured on PS fibrillar scaffolds and culture dish after staining with calcein-AM (green; live cell labelling) and ethidium homodimer-1 (red; dead cell labelling) at 3 DIV. Scale bar denotes 20 μm. (b) Statistical analysis of neuronal cell viability on various PS substrates at 3 and 7 DIV.

**
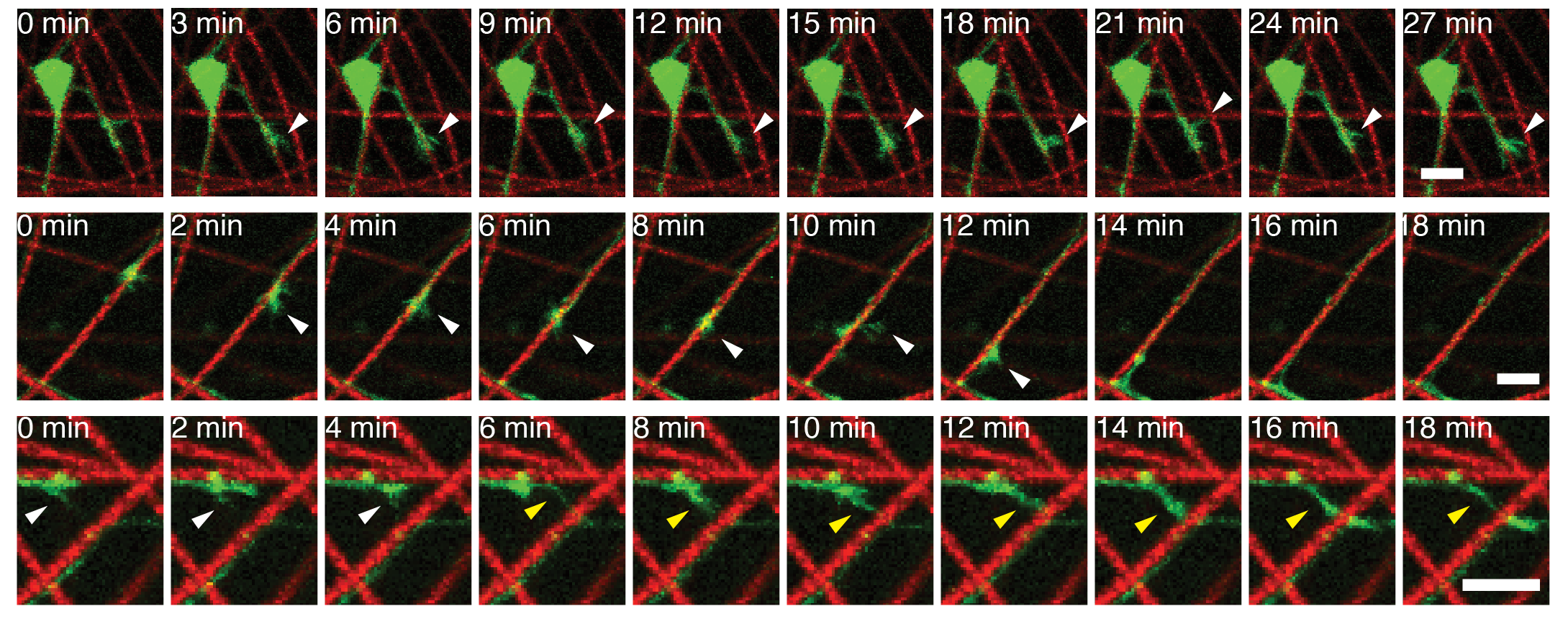
**

**Figure S2**. Time-lapse images of a 3 DIV neuron cultured on a electrospun fibrillar scaffold. (green: actin, red: PS fiber, all scale bars: 10 μm)


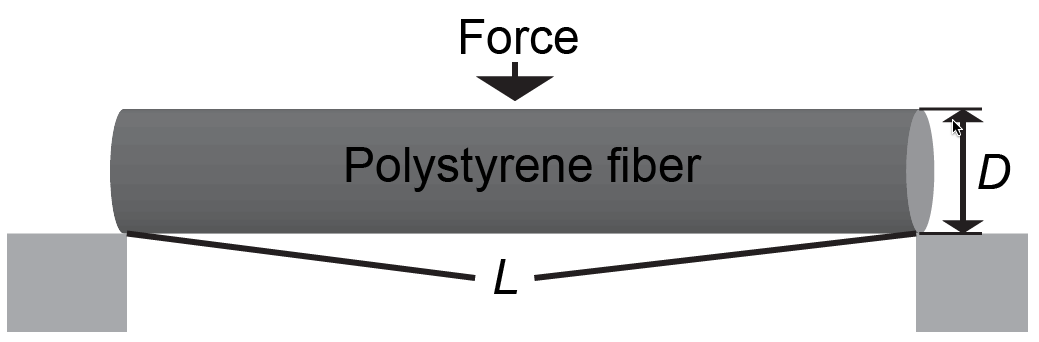


**Figure S3**. A schematic of theoretical calculation of fiber stiffness, which is proportional to , where E, D and L are Young’s modulus, diameter, and length of fiber, respectively.


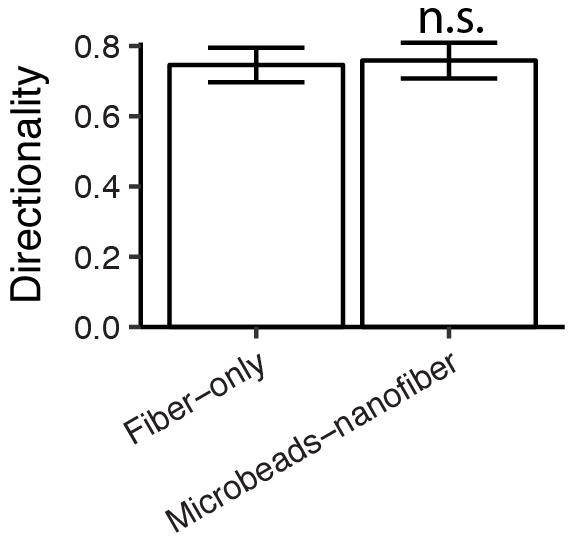


**Figure S4.** Comparison of neurite directionality in 5-DIV neuron cultures on fiber-only and microbead-nanofiber scaffolds. Fiber-only scaffolds with the average diameter of 2.2 μm at the medium fiber density were employed in this plot (n.s = 0.86, n = 15 for each scaffold).


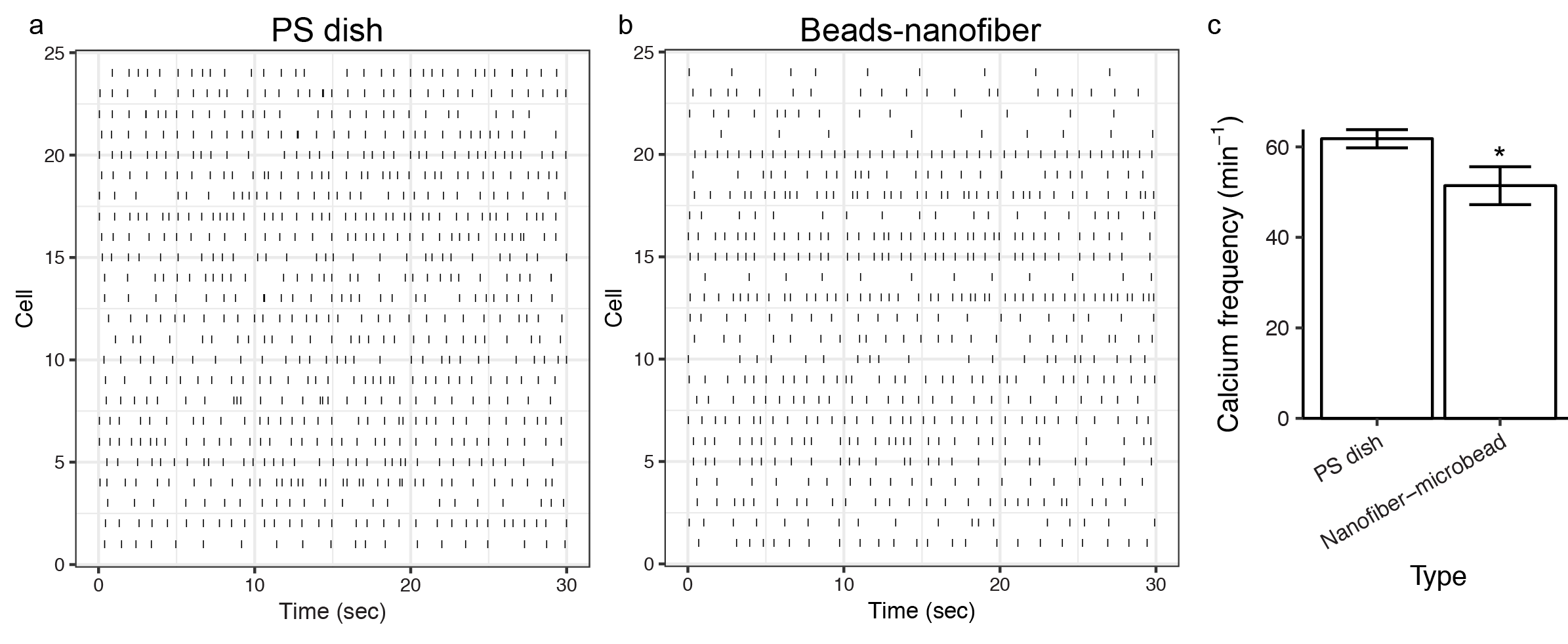


**Figure S5**. Raster plots of neuronal calcium signaling (at 14 DIV) on PDL-coated (a) PS dish and (b) PS bead-nanofiber scaffold. (c) Statistical distributions of calcium signal frequency on PS dish and microbead-nanofiber scaffold (*P < 0.05, n = 25 for each scaffold).


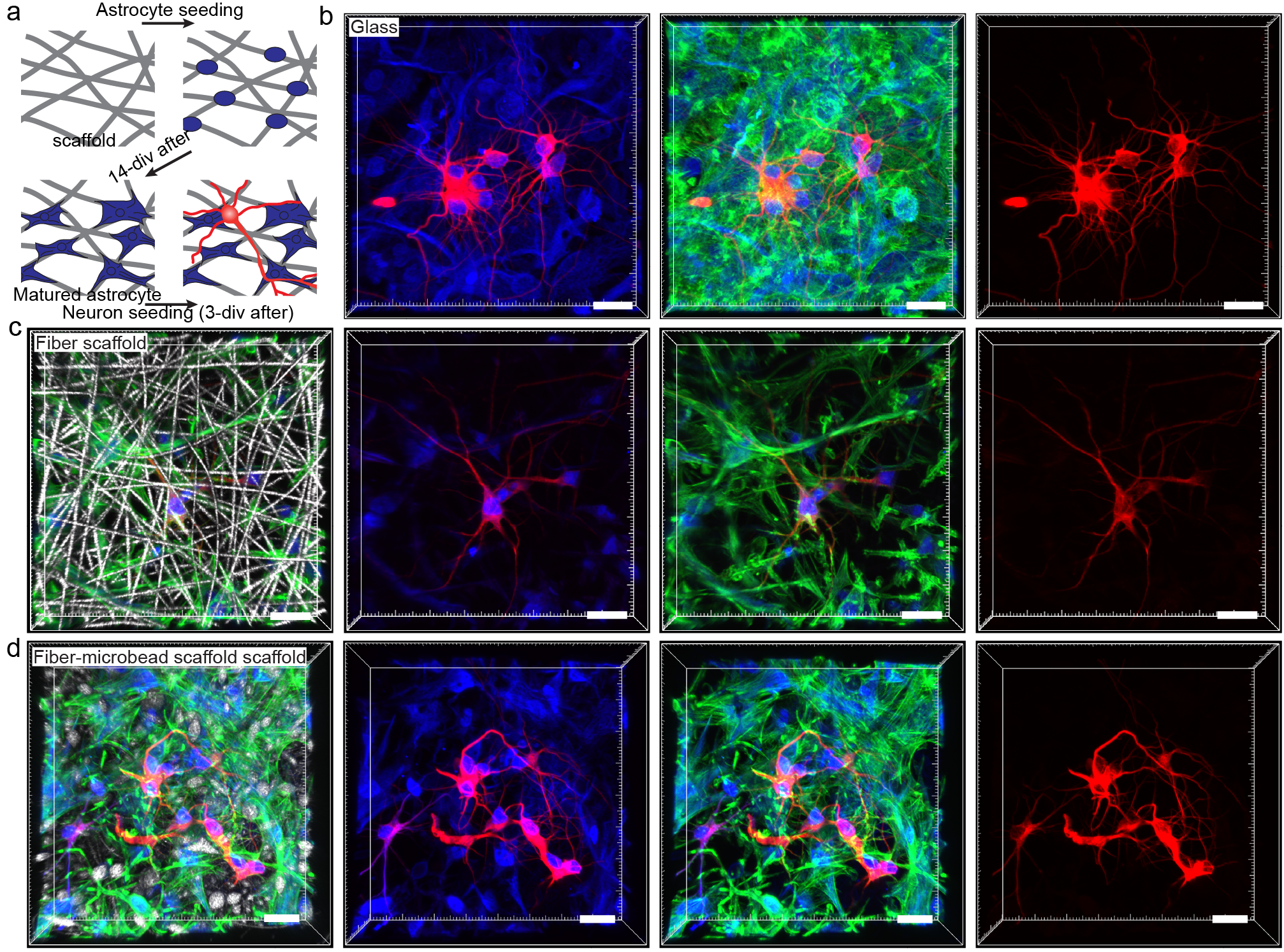


**Figure S6**. The 3D reconstructed fluorescent images of the co-culture of astrocytes and neurons cultivated on glass, PS fiber only, and PS nanofiber-microbead scaffolds. (a) A schematic description of co-culture procedures. Initially, the postnatal (3 DIV) astrocytes were cultivated for two weeks for proliferation, followed by the deposition of the prenatal (18 DIV) neurons on top. (b) The co-culture of neurons (3 DIV) and astrocytes (17 DIV) on glass. (c) The co-culture of astrocytes and neurons on the PS fibrillar scaffold. (d) The co-culture of astrocytes and neurons on the PS nanofiber-microbead scaffold (white: electrospun fibers/beads, red: neurons labeled with tuj1, blue: astrocytes labeled with GFAP, green: actin labeled with phalloidin 488). All images were three-dimensionally reconstructed from the collective stack of sectioned images taken with laser scanning confocal microscope (FV-1000, Olympus). Scale bars denote 20 μm.


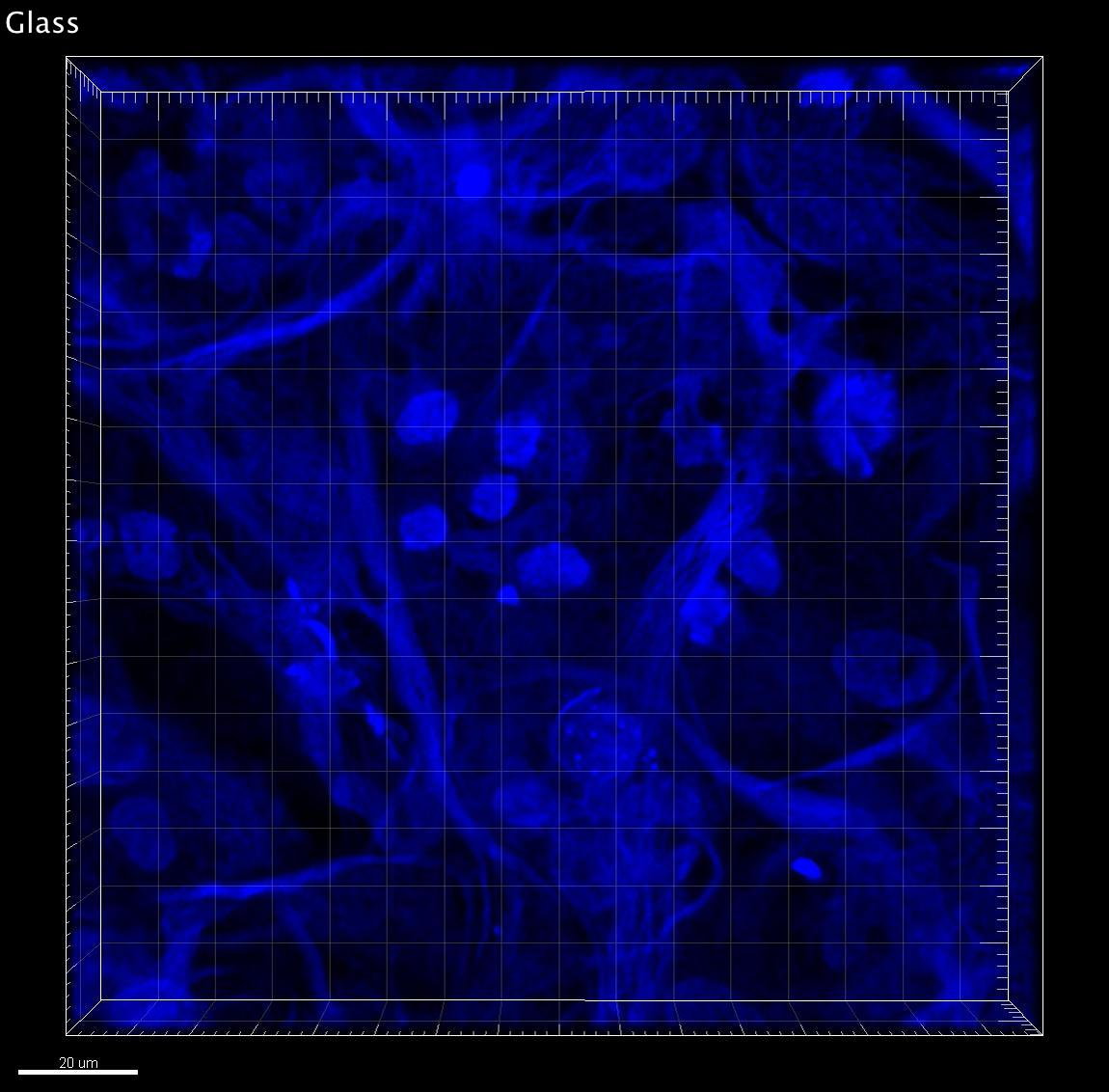


**Movie S1.** The 3D reconstruction of the co-culture of neurons and astrocytes cultivated on a glass substrate (white: electrospun fibers/beads, red: neurons labeled with tuj1, blue: astrocytes labeled with GFAP, green: actin labeled with phalloidin 488). The image was three-dimensionally reconstructed from the collective stack of sectioned images taken with laser scanning confocal microscope (FV-1000, Olympus). Scale bars denote 20 μm.


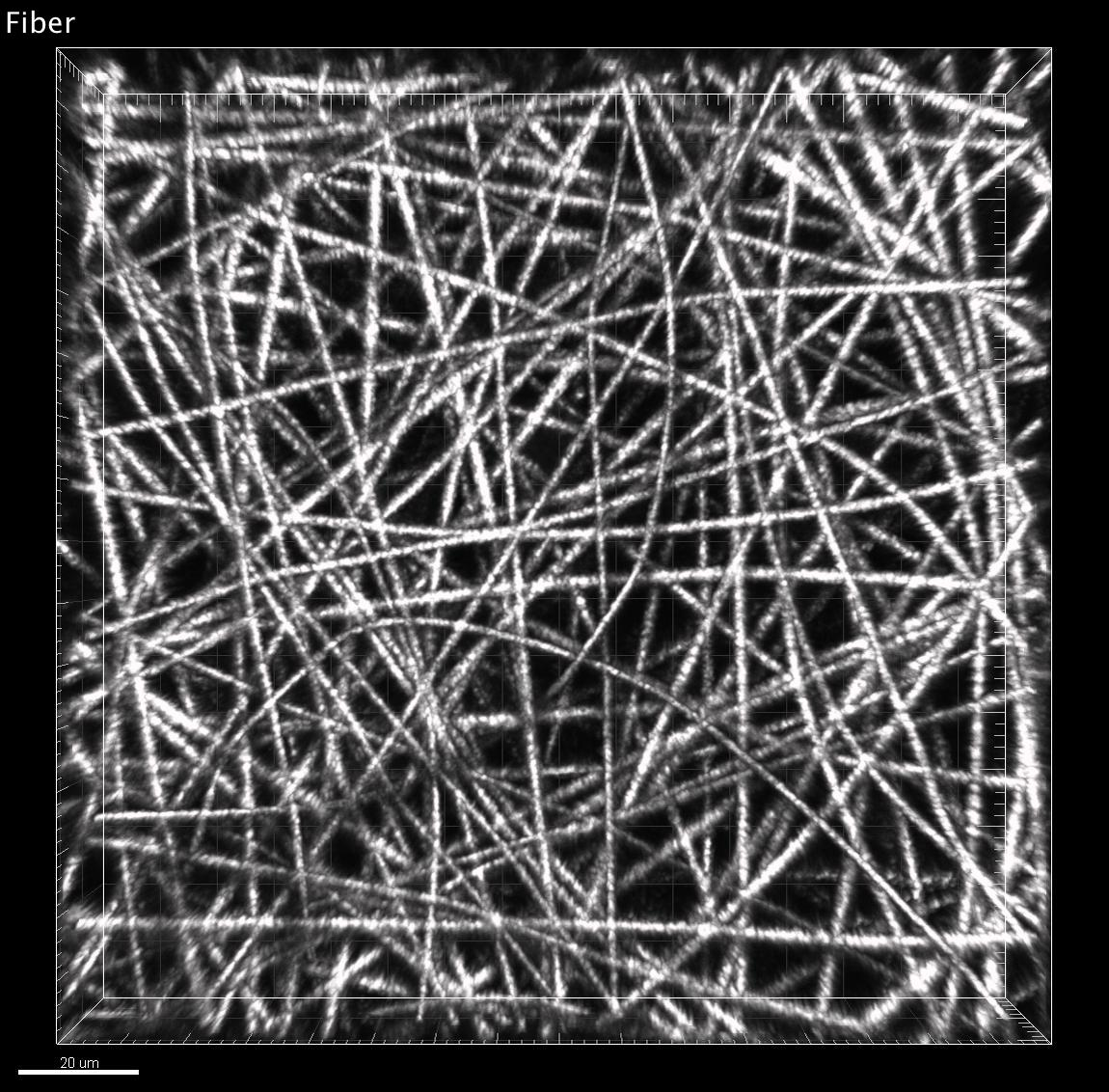


**Movie S2.** The 3D reconstruction of the co-culture of neurons and astrocytes cultivated on the PS fiber-only scaffold (white: electrospun fibers/beads, red: neurons labeled with tuj1, blue: astrocytes labeled with GFAP, green: actin labeled with phalloidin 488). The image was three-dimensionally reconstructed from the collective stack of sectioned images taken with laser scanning confocal microscope (FV-1000, Olympus). Scale bars denote 20 μm.


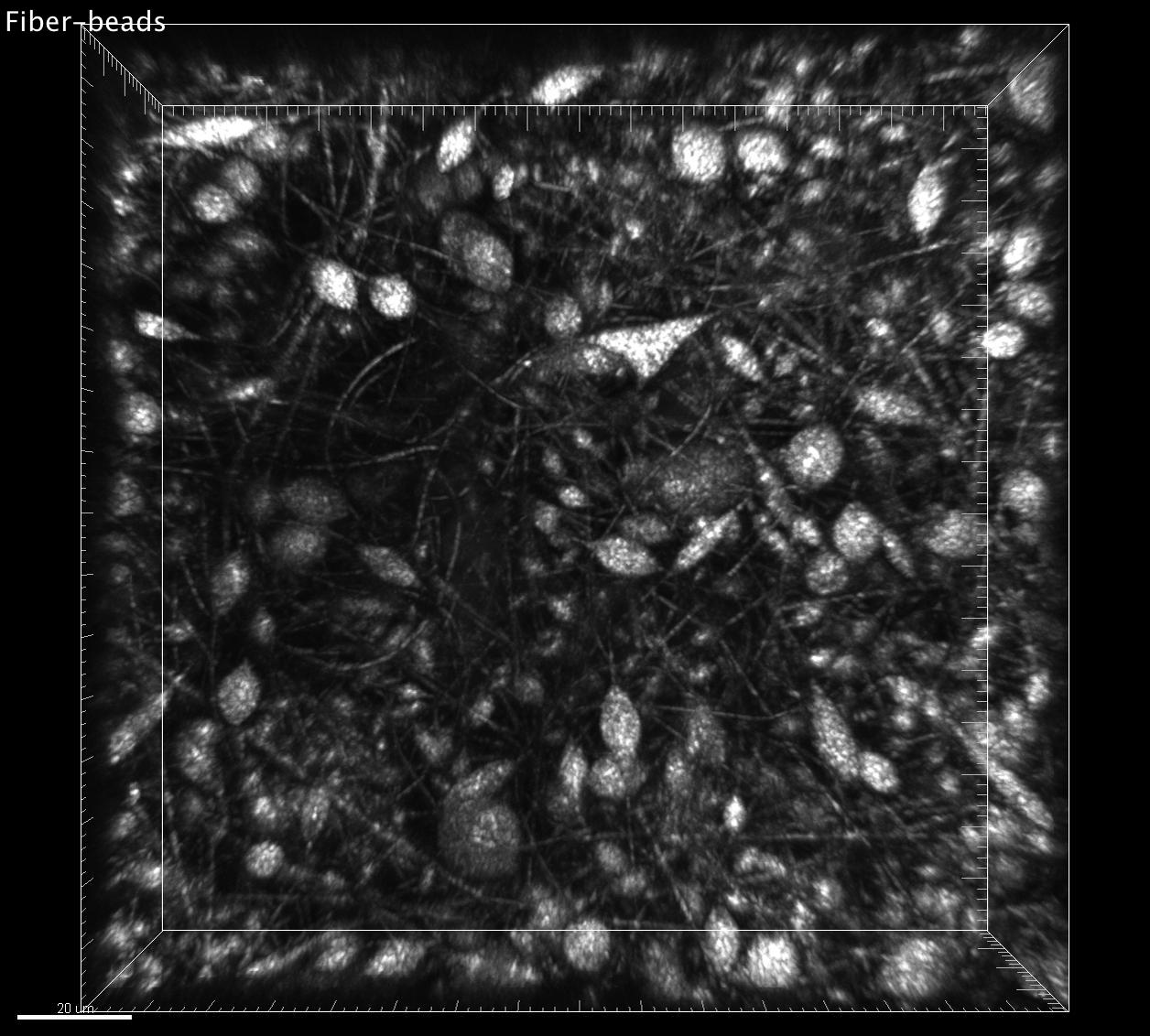


**Movie S3.** The 3D reconstruction of the co-culture of neurons and astrocytes cultivated on the PS nanofiber-bead scaffold (white: electrospun fibers/beads, red: neurons labeled with tuj1, blue: astrocytes labeled with GFAP, green: actin labeled with phalloidin 488). The image was three-dimensionally reconstructed from the collective stack of sectioned images taken with laser scanning confocal microscope (FV-1000, Olympus). Scale bars denote 20 μm.
